# Global analysis of gene expression in mineralizing fish vertebra-derived cell lines: new insights into anti-mineralogenic effect of vanadate

**DOI:** 10.1186/1471-2164-12-310

**Published:** 2011-06-13

**Authors:** Daniel M Tiago, Vincent Laizé, Luca Bargelloni, Serena Ferraresso, Chiara Romualdi, M Leonor Cancela

**Affiliations:** 1 Centre of Marine Sciences (CCMAR), University of Algarve, Faro, Portugal; 2 Department of Public Health, Comparative Pathology and Veterinary Hygiene, University of Padova, Legnaro, Italy; 3 CRIBI, University of Padova, Complesso Biologico Vallisneri, Via Ugo Bassi 58/B, Padova, Italy; 4 Department of Biomedical Sciences and Medicine, University of Algarve, Faro, Portugal

## Abstract

**Background:**

Fish has been deemed suitable to study the complex mechanisms of vertebrate skeletogenesis and gilthead seabream (*Sparus aurata*), a marine teleost with acellular bone, has been successfully used in recent years to study the function and regulation of bone and cartilage related genes during development and in adult animals. Tools recently developed for gilthead seabream, *e.g. *mineralogenic cell lines and a 4 × 44K Agilent oligo-array, were used to identify molecular determinants of *in vitro *mineralization and genes involved in anti-mineralogenic action of vanadate.

**Results:**

Global analysis of gene expression identified 4,223 and 4,147 genes differentially expressed (fold change - FC > 1.5) during *in vitro *mineralization of VSa13 (pre-chondrocyte) and VSa16 (pre-osteoblast) cells, respectively. Comparative analysis indicated that nearly 45% of these genes are common to both cell lines and gene ontology (GO) classification is also similar for both cell types. Up-regulated genes (FC > 10) were mainly associated with transport, matrix/membrane, metabolism and signaling, while down-regulated genes were mainly associated with metabolism, calcium binding, transport and signaling. Analysis of gene expression in proliferative and mineralizing cells exposed to vanadate revealed 1,779 and 1,136 differentially expressed genes, respectively. Of these genes, 67 exhibited reverse patterns of expression upon vanadate treatment during proliferation or mineralization.

**Conclusions:**

Comparative analysis of expression data from fish and data available in the literature for mammalian cell systems (bone-derived cells undergoing differentiation) indicate that the same type of genes, and in some cases the same orthologs, are involved in mechanisms of *in vitro *mineralization, suggesting their conservation throughout vertebrate evolution and across cell types. Array technology also allowed identification of genes differentially expressed upon exposure of fish cell lines to vanadate and likely involved in its anti-mineralogenic activity. Many were found to be unknown or they were never associated to bone homeostasis previously, thus providing a set of potential candidates whose study will likely bring insights into the complex mechanisms of tissue mineralization and bone formation.

## Background

Vertebrate skeleton is a multifunctional organ providing protection for soft tissues, structural support for muscle and connective tissues, storage for calcium and phosphorus, and a site for haematopoietic cell production and B lymphocyte maturation in adults [1]. Skeletogenesis, *i.e. *skeleton formation during development, is a process involving complex cellular and molecular mechanisms associated with ossification and bone remodeling. Both processes are known to be responsible for maintaining bone mass and skeletal integrity throughout life [2]. Skeletogenesis requires concerted interplay between various cellular activities (*e.g*. osteoblast and chondrocyte differentiation [1]) and numerous molecular determinants (*e.g. *skeletal proteins, growth factors, transcriptional regulators and signaling pathways [2]). Although human and mouse genetics have greatly contributed to unveil the mechanisms involved in skeletogenesis, information remains often insufficient for the successful development of therapies targeting skeletal diseases. Recent studies, reviewed by McGonnell and Fowkes [3], have demonstrated the suitability of fish models to investigate vertebrate development and in particular skeletogenesis. The resemblance of biochemical and physiological processes from fish to mammals, the presence in fish of orthologs for most mammalian genes, the similarities in organ morphology and systems composition are among the traits that contributed to the recent and rapid interest in fish models. Those combined with technical advantages, *e.g. *large progeny, external reproduction fast growth and translucent larvae, and the power of fish genetics, have definitively transformed fish systems as promising alternatives to mammalian systems. In fact, various fish mutants have already been used to model human skeletal diseases: osteogenesis imperfecta (an autosomal dominant disorder characterized by extreme bone fragility) can be modeled by zebrafish *chihuahua *mutant [4]; craniofacial syndromes (holoprosencephaly, campomelic dysplasia and Ehlers-Danlos syndrome) can be modeled by zebrafish *sonic you*, *jellyfish*, and *b4galt7 *mutants [5,6], idiopathic scoliosis can be modeled by guppy *curve back *mutant [7] and important efforts have consequently been made towards the development of fish biochemical, molecular, and cellular tools [8]. These include: (i) the sequence of genomes of various fish models, *e.g. *zebrafish, green-spotted puffer fish, Japanese medaka and stickleback; (ii) the development of large collections of expressed sequence tags (EST) which were produced for several fish species (*e.g. *Atlantic salmon [9], rainbow trout [10], Atlantic halibut [11] or channel and blue catfish [12]); (iii) the development of several microarray platforms to explore these EST collections (*e.g. *Nimblegen Technology high-density oligo-array for the catfish [13], parallel synthesis technology high-density DNA microarray for the Atlantic halibut [14], Agilent SurePrint™ Technology oligo-array for the largemouth bass [15], the rainbow trout [16], and the gilthead seabream *Sparus aurata *[17]); and (iv) the development of several fish-derived cell lines (the Fish Cell Line Database, http://www.fcma.ualg.pt/edge/FICELdb.mht). To investigate mechanisms of tissue mineralization, various bone-derived cell lines of fish origin are available [18] and gilthead seabream VSa13 and VSa16 cell lines (derived from vertebra) are of particular interest due to their pre-chondrocyte and pre-osteoblast phenotypes. Although they are both capable of mineralizing their extracellular matrix, VSa13 and VSa16 cell lines behave differently regarding their degree of mineral deposition [19], levels of alkaline phosphatase activity [19], expression of mineralogenic genes, *e.g. matrix gla protein (MGP), osteocalcin (OC), osteopontin (SPP1), bone morphogenetic protein-2 (BMP-2) *[19-21], and susceptibility to mineralogenic or anti-mineralogenic molecules, *e.g. *insulin, IGF-1, vanadate [22,23] and retinoic acid [unpublished results]. They are therefore considered as different bone cell types and common genes differentially expressed during *in vitro *mineralization should represent key mineralogenic genes, while other differentially expressed genes would represent genes involved in cell type-specific processes not related to mineralization. In this work, we have used an Agilent Sureprint 4 × 44K oligo-array, containing two non-overlapping probes for each of 19,734 unique gene transcripts [17], to analyze global gene expression during *in vitro *mineralization of these two cell lines. We have also identified in VSa13 cell line the presence of mineralogenic genes whose expression was altered upon cell exposure to vanadate, a molecule with anti-mineralogenic activity [23].

## Results

### Genes differentially expressed during *in vitro *mineralization of gilthead seabream vertebra-derived cell lines

**Figure 1:**
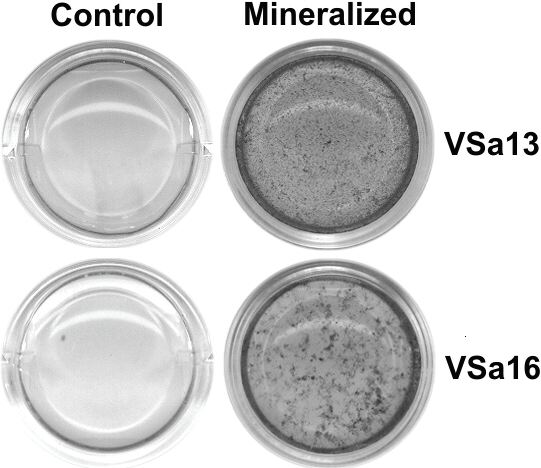
**Pictures of pre-chondrocyte (VSa13) and pre-osteoblast (VSa16) cells undergoing mineralization**. Cultures were treated for 4 weeks under control or mineralizing conditions then von Kossa stained to reveal mineral deposition.

**Figure 2:**
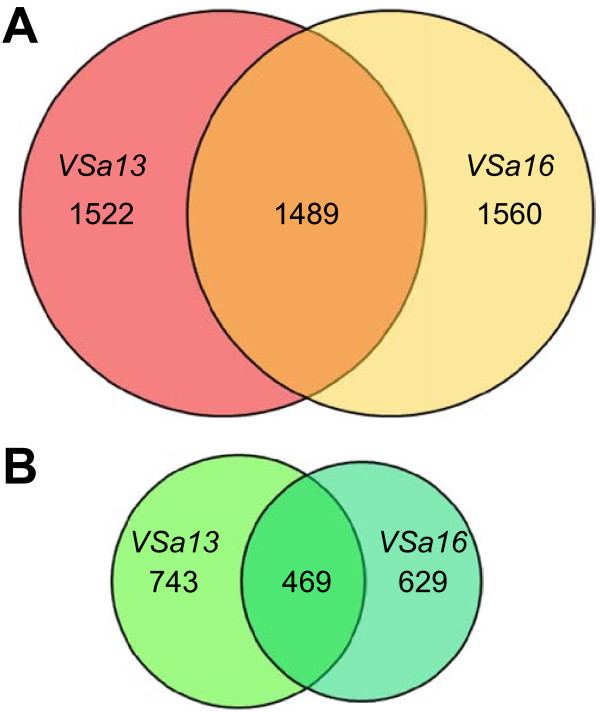
**Venn diagrams of genes up-regulated (A) and down-regulated (B) during mineralization of VSa13 and VSa16 cells**. A two class SAM test was performed; FDR and FC parameters were lower than 5% and higher than 1.5, respectively. Size of diagrams is proportional to the size of gene pools.

Confluent cultures of VSa13 and VSa16 cells were cultivated during 4 weeks under control (regular medium) or mineralizing (regular medium supplemented with calcium, phosphate and L-ascorbic acid) conditions. Deposition of mineral nodules within extracellular matrix was confirmed by von Kossa staining in cells exposed to mineralization cocktail (Figure 1) and total RNA was extracted from three biological replicates per condition. After proper amplification and labeling, each RNA sample was hybridized against the oligo-array and raw expression data were extracted and filtered using Agilent Feature Extraction 9.5.1 software, then normalized. Quantile normalization showed the highest agreement among replicates (*i.e. *lowest variation of normalized fluorescence distribution among replicates; data not shown) and was consequently used to normalize raw data sets. Normalized data sets were analyzed through significance analysis of microarray (SAM) and genes differentially expressed in control *versus *mineralizing conditions were identified. False discovery rate (FDR) threshold was set at 5% and only probes with fold change (FC) over 1.5 were considered. A total of 4,777 and 4,554 probes - corresponding to 3,011 and 3,049 unique genes - indicative of an up-regulated expression were identified from VSa13 and VSa16 RNAs, respectively. Among these genes, 1,489 were shown to be common to both cell lines (Figure 2). Similarly, 2,359 and 1,642 probes - corresponding to 1,212 and 1,098 unique genes - indicative of a down-regulated expression were identified from VSa13 and VSa16 RNAs, respectively. Among these genes, 469 were shown to be common to both cell lines (Figure 2). In VSa13 and VSa16 cells, 69% and 49% of differentially expressed genes were simultaneously detected by two non-overlapping probes, respectively, which could indicate the high occurrence of alternative splicing. It could also be the consequence of slight differences of hybridization between the two probes. Indeed, most genes detected by only one probe seem to follow the same pattern of expression when compared to the second non-significant probe. Raw and normalized fluorescence data have been deposited in the GEO database under accession numbers GSE18915 (VSa13) and GSE18941 (VSa16).

### Ontology of mineralization-related genes

**Figure 3:**
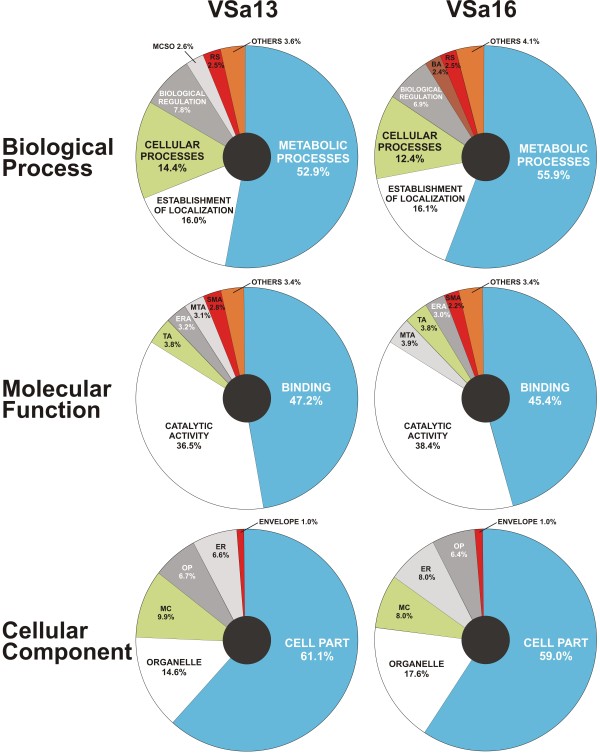
**Pie chart representations of GO entries occurrence among genes detected in VSa13 and VSa16 cells**. Pie charts represent biological processes, molecular function and cellular component GO entries occurrence among differentially expressed genes in control *versus *mineralized VSa13 and VSa16 cells. Raw data were normalized using the quantile method and a two class SAM test was performed; FDR and FC were lower than 5% and higher than 1.5, respectively. Biological processes GO entries were associated with 19.2% (VSa13) and 16.6% (VSa16) of identified genes; molecular function GO entries were associated with 24.3% (VSa13) and 20.7% (VSa16) of identified genes; and cellular component GO entries were associated to 18.5% (VSa13) and 10.3% (VSa16) of identified genes. GO definitions: BA, biological adhesion; ER, extracellular region; ERA, enzyme regulator activity; MC, macromolecular complex; MCSO, macromolecular complex subunit organization; MTA, molecular transducer activity; OP, organelle part; RS, response to stimulus; SMA, structural molecule; TA, transporter activity. Additional information on GO definition is available in Additional files 1, 2 and 3, Tables S1, S2 and S3.

Genes differentially expressed during *in vitro *mineralization were classified according to their putative Gene Ontology (GO) - biological process (BP), molecular function (MF) and cellular component (CP) - using OBO-Edit software, AmiGO database [24] and SAPD database [25], based on significant similarity with known genes in public data bases. In general, a GO term could be associated with less than 25% of mineralization-related genes (% of BP/MF/CP was 19.2/24.3/18.5 in VSa13 cells and 16.6/20.7/10.3 in VSa16 cells). Comparative analysis of GO classifications indicated that the same type of genes, but not necessary the same genes, were involved in extracellular matrix (ECM) mineralization of both cell lines (Figure 3). Moreover, similar occurrence of BP, MF and CP classes was observed in genes common to both cell lines (Additional files 1, 2 and 3, Tables S1, S2 and S3). The most represented BP classes (Figure 3A and Additional file 1, Table S1) were: (i) metabolism (52.9% and 55.9% in VSa13 and VSa16 cells, respectively), (ii) establishment of localization (16% and 16.1%; mostly related to transport), (iii) cellular processes (14.4% and 12.4%, mostly related to signaling) and (iv) regulation (7.8% and 6.9%, mostly associated to cell cycle). Most MF classes were related to binding activity (47.2% and 45.4%; mostly to nucleotides, ions and nucleic acid) and catalytic activity (36.5% and 38.4%) (Figure 3B and Additional file 2, Table S2). A smaller part was involved in transport, enzyme regulation and molecular transduction activities (total of 10.1% and 10.7% in VSa13 and VSa16 cells, respectively). Finally, most CP classes were related to cytosol or membrane compartments (61.1% and 59.0%) and to specific organelles (14.6% and 17.6%; nucleus, endoplasmic reticulum or Golgi complex). A smaller fraction was associated to macromolecular complexes (9.9% and 8.0%), extracellular region (6.6% and 8.0%) and organelle parts (6.7% and 6.4%). In a general manner, the identification of numerous and diverse genes in both cell lines suggested that ECM mineralization is a complex process that requires tight regulatory mechanisms. In order to understand which type of differentially expressed genes were enriched in these cell lines, we performed a functional annotation analysis using the **D**atabase for **A**nnotation, **V**isualization and **I**ntegrated **D**iscovery (DAVID v6.7, available at http://david.abcc.ncifcrf.gov[26,27]) and focused on processes with fold enrichment > 1.1 and significance p-value < 0.05. In both cell lines, results indicated an enrichment of genes associated with biosynthetic processes, cell cycle and growth, signaling, protein synthesis, stress response, biological regulation and metabolism (Table 1). Other processes such as protein interaction, DNA/RNA metabolism, development, adhesion and bone, were cell type-specific (only identified in VSa16 cells), confirming that although both cell lines are mineralogenic, they also represent different bone cell types.

**Table 1:** List of biological processes GO categories enriched among genes differentially expressed in mineralizing VSa13 and VSa16 cells

	VSa13 (CHONDROCYTIC LINEAGE)					VSa16 (OSTEOBLASTIC LINEAGE)				
***BP***	***TERM***	***COUNT***	***%***	***P***	***FE***	***TERM***	***COUNT***	***%***	***P***	***FE***

						protein homotetramerization	6	0.5	0.006	3.8
PROT. INTER.						protein tetramerization	7	0.5	0.013	2.9
						protein homooligomerization	9	0.7	0.024	2.3

						rRNA transcription	5	0.4	0.019	3.8
DNA/RNA METAB.						DNA replication	37	2.9	0.000	2.1
						DNA-dependent DNA replication	19	1.5	0.001	2.1

	serine family amino acid biosynt. process	6	0.4	0.025	2.9	vitamin biosynthetic process	10	0.8	0.003	2.7
	hexose/alcohol/monosaccharide biosynthetic process	12	0.8	0.005	2.3	water-soluble vitamin biosynthetic process	9	0.7	0.008	2.6
BIOSYN. PROC.	amino acid biosynthetic process	15	1.0	0.043	1.7	hexose/alcohol/monosaccharide biosynthetic process	10	0.8	0.028	2.1
	carbohydrate biosynthetic process	21	1.5	0.016	1.6	coenzyme biosynthetic process	21	1.6	0.012	1.7
	nitrogen compound biosynthetic process	21	1.5	0.027	1.6	cofactor biosynthetic process	24	1.9	0.035	1.5
	amine biosynthetic process	19	1.3	0.040	1.5					

						pigmentation	7	0.5	0.026	2.6
						inner ear morphogenesis	7	0.5	0.045	2.4
						fertilization	9	0.7	0.024	2.3
DEV.						tissue remodeling	14	1.1	0.009	2.0
						embryonic morphogenesis	14	1.1	0.013	2.0

						tissue development	33	2.5	0.005	1.6
						embryonic development	33	2.5	0.010	1.5
	mitochondrion organization and biogenesis	15	1.0	0.016	1.8	mitotic cell cycle checkpoint	9	0.7	0.024	2.3
	cell motility/localization of the cell	59	4.1	0.002	1.4	cell cycle checkpoint	12	0.9	0.015	2.1
	mitosis/M phase of mitotic cell cycle	35	2.4	0.024	1.4	regulation of mitosis	14	1.1	0.009	2.0
	cell projection: morphogenesis,	28	2.0	0.040	1.4	growth	38	2.9	0.001	1.6
CELL CYCLE	organization and biogenesis					regulation of cell growth	18	1.4	0.042	1.6
AND	M phase	40	2.8	0.034	1.3	regulation of growth	25	1.9	0.018	1.5
GROWTH	cell division	36	2.5	0.041	1.3	cell growth	24	1.9	0.023	1.5
	cell proliferation	89	6.2	0.013	1.2	regulation of cell size	24	1.9	0.035	1.5
						cell motility/localization of the cell	52	4.0	0.007	1.4
						mitosis/M phase of mitotic cell cycle	33	2.5	0.018	1.4
						cell migration	31	2.4	0.027	1.4
						mitotic cell cycle	43	3.3	0.039	1.3
						cell proliferation	78	6.0	0.047	1.2

SIGNAL.	DNA damage response. signal transduction resulting in induction of apoptosis	5	0.3	0.028	3.4	phosphoinositide-mediated signaling	16	1.2	0.010	1.9

PROT.	tRNA modification	6	0.4	0.010	3.4	tRNA aminoacylation for protein translation/aminoacid activation	14	1.1	0.025	1.8
SYNT.	RNA modification	9	0.6	0.001	3.1					

	response to virus	14	1.0	0.003	2.2	response to wounding	50	3.9	0.006	1.4
STRESS	cell redox homeostasis	17	1.2	0.003	2.0	inflammatory response	32	2.5	0.049	1.4
RESP.	response to oxidative stress	19	1.3	0.040	1.5	response to stress	112	8.6	0.028	1.2
	response to stimulus	216	15.1	0.011	1.1	response to stimulus	194	15.0	0.026	1.1

ADH.						biological/cell adhesion	65	5.0	0.026	1.3

BIOL. REG.	regulation of biological quality	103	7.2	0.018	1.2	regulation of biological quality	97	7.5	0.008	1.2

	isoprenoid metabolic process	9	0.6	0.015	2.4	purine ribonucleoside metabolic process	5	0.4	0.019	3.8
	glutathione metabolic process	8	0.6	0.033	2.3	ribonucleoside/glycine metabolic process	6	0.5	0.017	3.2
	pyruvate metabolic process	11	0.8	0.017	2.1	retinoid/diterpenoid metabolic process	5	0.4	0.046	3.1
	gluconeogenesis	9	0.6	0.043	2.1	aldehyde metabolic process	6	0.5	0.035	2.8
	mitochondrial transport	12	0.8	0.032	1.9	serine family amino acid metabolic process	12	0.9	0.002	2.5
	sulfur metabolic process	17	1.2	0.010	1.8	glutamine family amino acid metab. process	12	0.9	0.004	2.4
	water-soluble vitamin metabolic process	13	0.9	0.049	1.7	nucleoside/pyridine nucleotide metab. process	10	0.8	0.011	2.4
	glucose metabolic process	26	1.8	0.010	1.6	water-soluble vitamin metabolic process	16	1.2	0.001	2.3
	glucose catabolic process	17	1.2	0.037	1.6	gluconeogenesis	9	0.7	0.024	2.3
	vitamin/alcohol metabolic process	18	1.3	0.039	1.6	oxygen and ROS metabolic process	8	0.6	0.033	2.3
	nucleotide metabolic process	47	3.3	0.001	1.5	vitamin metabolic process	23	1.8	0.000	2.2
	nucleobase. nucleoside and nucleotide metabolic process	49	3.4	0.002	1.5	pyruvate metabolic process	10	0.8	0.028	2.1
	monosaccharide metabolic process	32	2.2	0.016	1.5	glucose metabolic process	26	2.0	0.002	1.8
	carboxylic/organic acid metab. process	102	7.1	0.000	1.4	glucose catabolic process	17	1.3	0.015	1.8
	amino acid and derivative metabolic process	67	4.7	0.002	1.4	amino acid catabolic process	15	1.2	0.025	1.8
	amino acid metabolic process	54	3.8	0.008	1.4	alcohol catabolic process	18	1.4	0.015	1.7
	monocarboxylic acid metabolic process	45	3.1	0.015	1.4	hexose/monosaccharide catabolic process	17	1.3	0.026	1.7
	hexose metabolic process	31	2.2	0.023	1.4	amine/nitrogen compound catabolic process	16	1.2	0.034	1.7
	fatty acid metabolic process	31	2.2	0.034	1.4	monosaccharide metabolic process	31	2.4	0.007	1.6
METAB.	lipid biosynthetic process	32	2.2	0.040	1.4	aromatic compound metabolic process	23	1.8	0.012	1.6
	amine metabolic process	74	5.2	0.006	1.3	carbohydrate catabolic process	21	1.6	0.025	1.6
	nitrogen compound metabolic process	75	5.2	0.010	1.3	tRNA metabolic process	19	1.5	0.026	1.6
	alcohol metabolic process	47	3.3	0.021	1.3	cellular carbohydrate catabolic process	20	1.5	0.032	1.6
	cellular carbohydrate metabolic process	52	3.6	0.036	1.3	amino acid metabolic process	54	4.2	0.001	1.5
	catabolic process	106	7.4	0.012	1.2	nucleobase. nucleoside and nucleotide metabolic process	46	3.5	0.001	1.5
	lipid metabolic process	85	5.9	0.012	1.2	nucleotide metabolic process	42	3.2	0.003	1.5
	cellular lipid metabolic process	69	4.8	0.037	1.2	coenzyme metabolic process	37	2.9	0.011	1.5
						hexose metabolic process	30	2.3	0.011	1.5
						carboxylic/organic acid metabolic process	97	7.5	0.000	1.4
						amine metabolic process	72	5.6	0.001	1.4
						amino acid and derivative metabolic process	62	4.8	0.002	1.4
						nitrogen compound metabolic process	73	5.6	0.002	1.4
						cofactor metabolic process	46	3.5	0.007	1.4
						cellular carbohydrate metabolic process	51	3.9	0.009	1.4
						carbohydrate metabolic process	67	5.2	0.009	1.3
						alcohol metabolic process	43	3.3	0.026	1.3
						cellular catabolic process	84	6.5	0.017	1.2
						catabolic process	95	7.3	0.027	1.2
						lipid metabolic process	75	5.8	0.037	1.2
						cellular lipid metabolic process	63	4.9	0.043	1.2

						bone mineralization	6	0.5	0.035	2.8
BONE						ossification/biomineral formation/bone remodelling	13	1.0	0.011	2.0
						skeletal development	28	2.2	0.009	1.6

Functional annotation tool from the **d**atabase for **a**nnotation, **v**isualization and **i**ntegrated **d**iscovery (DAVID; v6.7) has been used to identify significant biological processes among differentially expressed genes. p < 0.05 (P) and fold enrichment (FE) was higher than 1.1.

In an attempt to pinpoint the most relevant mineralogenic genes, we looked at genes with a FC > 10 (FDR was maintained to < 5%). A total of 46/48 up-regulated and 49/49 down-regulated genes were identified in VSa13 and VSa16 cells, respectively. Most of them (61% in VSa13 cells and 59% in VSa16 cells) did not match any known gene (Additional files 4, 5, 6 and 7, Tables S4, S5, S6 and S7). Of these genes, 33 were common to both cell lines, GO analysis indicated that up-regulated genes were mainly associated with (i) transport, (ii) matrix/membrane, (iii) metabolism and (iv) signaling, while down-regulated genes were mainly associated with (i) metabolism, (ii) calcium binding, (iii) transport and (iv) signaling.

### Proliferative and anti-mineralogenic effects of vanadate: identification of genes with significant patterns of expression

**Figure 4:**
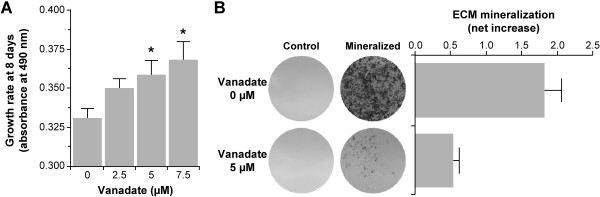
**Effect of vanadate on VSa13 cell proliferation (A) and ECM mineralization (B)**. For proliferation experiments VSa13 cells were seeded in 96-well plates at 1.5 × 10^3 ^cells/well then either left untreated or treated with 2.5, 5 and 7.5 μM of vanadate. Cell proliferation was evaluated after 8 days using MTS assay. For ECM mineralization experiments VSa13 cells were seeded in 24-well plates at 2 × 10^4 ^cells/well, grown until confluence then treated for mineralization. Mineralizing cultures were then either left untreated or treated with 5 μM vanadate. Mineral deposition was revealed after 4 weeks by von Kossa staining and evaluated by densitometry analysis. Asterisk indicates values statistically different from corresponding controls (n ≥ 3; *P *< 0.05; one-way ANOVA).

**Figure 5:**
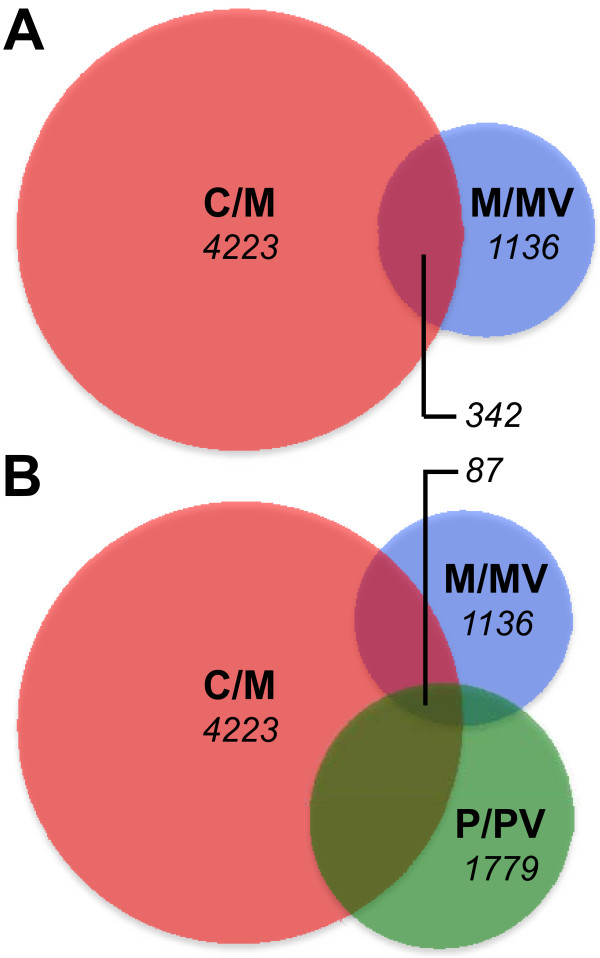
**Venn diagrams of differentially expressed genes in vanadate-treated VSa13 cells**. (A) Genes detected in mineralizing cells (C/M) were compared with genes detected in mineralizing cells treated with vanadate (M/MV). (B) Genes detected in C/M were compared with genes detected in M/MV and with genes detected in proliferating cells treated with vanadate (P/PV). Two-class SAM tests were performed with FDR and FC limits lower than 5% and higher than 1.5, respectively. Size of diagrams is proportional to the size of gene pools.

In order to further investigate genes involved in ECM mineralization, we exposed dividing and mineralizing VSa13 cells to vanadate, an ultra-trace metal with anti-mineralogenic effect in fish vertebra-derived cell lines, and analyzed global gene expression. Any gene differentially expressed upon vanadate treatments would be potentially important for differentiation/mineralization mechanisms. As expected from previous studies [23,28], cell proliferation was stimulated by vanadate at concentrations up to 7.5 μM, while ECM mineralization was inhibited by vanadate at concentrations up to 5 μM (Figure 4). Total RNA was collected from three biological replicates of proliferating and mineralizing VSa13 cells exposed to vanadate or left untreated. After proper amplification and labeling, each RNA sample was hybridized against gilthead seabream oligo-array and data were extracted, normalized, and analyzed as previously described. Differentially expressed genes were identified through a two-class SAM analysis with FDR < 5% and FC > 1.5 in the following data sets: i) control *versus *mineralization: 4,223 differentially expressed genes (3,011 up- and 1,212 down-regulated genes), ii) mineralization *versus *mineralization + vanadate: 1,136 differentially expressed genes (406 up- and 730 down-regulated genes), and iii) proliferation *versus *proliferation + vanadate: 1,779 differentially expressed genes (496 up- and 1283 down-regulated genes). Among those genes, 342 were common to conditions i) and ii) (Figure 5A). In order to identify key mineralogenic genes in VSa13 cells, we looked at genes which expression was oppositely regulated during *in vitro *mineralization and upon vanadate treatment. These genes and their respective GO categories are listed in Table 2 and could be classified according to a score calculated as the ratio between FC_M _(control *versus *mineralization) and FC_MV _(mineralization *versus *mineralization + vanadate). Most genes were shown to be associated with metabolism, cell matrix/adhesion, signaling and calcium binding. Fewer genes were associated with apoptosis, transport, proteolysis, structural activity, translation and growth.

**Table 2:** Gene description (according to SAPD database [28]), GO classification, FC and scores of common genes regulated during mineralization of VSa13 cells (FC_M_) *versus *mineralization with vanadate (FC_MV_)

Gene description	**GO**_**BP/MF**_	**FC**_**M**_	**FC**_**MV**_	Score
Photoreceptor outer segment all-trans retinol dehydrogenase [Source:IPIAcc:IPI00024598]	metabolic process/oxidoreductase activity	34,8	0,14	**240,1**

AMBP protein precursor [Source:Uniprot/SWISSPROTAcc:P02760]	-/-	47,4	0,36	**131,5**

Ependymin related protein-1 precursor [Source:IPIAcc:IPI00554718]	cell-matrix adhesion/calcium ion binding	15,2	0,14	**111,8**

Xaa-Pro aminopeptidase 2 precursor [Source:Uniprot/SWISSPROTAcc:O43895]	proteolysis, creatine metabolic process/metalloexopeptidase activity, hydrolase activity, creatinase activity	27,2	0,25	**110,2**

No match	-/-	28,2	0,31	**89,73**

Microtubule-associated proteins 1A/1B light chain 3C precursor [Source:Uniprot/SWISSPROTAcc:Q9BXW4]	-/-	12,6	0,14	**89,63**

Cell death activator CIDE-3 [Source:Uniprot/SWISSPROTAcc:Q96AQ7]	apoptosis/protein binding	7,77	0,17	**45,71**

No match	-/-	25,5	0,61	**41,50**

No match	-/-	12,7	0,32	**39,97**

PREDICTED: hypothetical protein [Danio rerio]	pore complex biogenesis/channel activity	18,3	0,52	**35,20**

Hypothetical protein LOC447879 [Source:RefSeq_peptideAcc:NP_001004618]	integrin-mediated signaling pathway/-	9,55	0,29	**32,64**

No match	-/-	17,5	0,54	**32,31**

Granulocyte-macrophage colony-stimulating factor receptor alpha chain precursor [Source:Uniprot/SWISSPROTAcc:P15509]	-/-	8,69	0,29	**29,83**

No match	-/-	5,77	0,21	**27,18**

IgGFc-binding protein precursor [Source:Uniprot/SWISSPROTAcc:Q9Y6R7]	cell adhesion/-	4,35	0,19	**23,12**

No match	-/-	6,28	0,28	**22,13**

Hydroxyacid oxidase 2 [Source:Uniprot/SWISSPROTAcc:Q9NYQ3]	metabolic process, electron transport/oxidoreductase activity	5,21	0,24	**21,38**

No match	-/-	7,70	0,39	**19,85**

No match	-/-	7,35	0,37	**19,79**

Phosphatase and actin regulator 4 isoform 1 [Source:RefSeq_peptideAcc:NP_001041648]	-/-	4,95	0,27	**18,08**

Ependymin related protein-1 precursor [Source:IPIAcc:IPI00554718]	cell-matrix adhesion/calcium ion binding	2,54	0,14	**18,02**

No match	-/-	8,95	0,52	**17,26**

No match	-/-	10,6	0,62	**17,00**

No match	-/-	6,53	0,39	**16,57**

Glucose-6-phosphate 1-dehydrogenase [Source:Uniprot/SWISSPROTAcc:P11413]	glucose metabolic process/glucose-6-phosphate dehydrogenase activity	4,30	0,27	**16,05**

Glutamate--cysteine ligase catalytic subunit [Source:Uniprot/SWISSPROTAcc:P48506]	-/-	4,71	0,29	**16,04**

Complement factor I precursor [Source:Uniprot/SWISSPROTAcc:P05156]	proteolysis/catalytic activity, serine-type endopeptidase activity, hydrolase activity, scavenger receptor activity	2,58	0,16	**15,91**

No match	-/-	5,66	0,41	**13,71**

No match	-/-	4,30	0,33	**12,95**

No match	-/-	3,64	0,29	**12,77**

No match	-/-	4,93	0,39	**12,73**

No match	-/-	5,41	0,43	**12,69**

Hypothetical protein LOC406276 [Source:RefSeq_peptideAcc:NP_998168]	-/calcium ion binding	5,07	0,40	**12,54**

Hypothetical protein LOC336637 [Source:RefSeq_peptideAcc:NP_956317]	cell redox homeostasis/-	4,66	0,38	**12,17**

Actin filament-associated protein 1-like 2. [Source:Uniprot/SWISSPROTAcc:Q8N4X5]	-/-	5,69	0,48	**11,79**

Transcribed locus, weakly similar to NP_175293.1 [Source:UniGeneAcc:Tru.931]	metabolic process/methyltransferase activity	2,82	0,24	**11,69**

No match	-/-	5,96	0,51	**11,66**

Ornithine carbamoyltransferase, mitochondrial precursor [Source:Uniprot/SWISSPROTAcc:P00480]	-/ornithine carbamoyltransferase activity, amino acid binding	4,17	0,37	**11,38**

No match	-/-	4,83	0,42	**11,38**

CDNA FLJ31025 fis, clone HLUNG2000501. [Source:Uniprot/SPTREMBLAcc:Q96ND9]	-/-	3,80	0,34	**11,25**

No match	-/-	3,71	0,34	**11,05**

No match	-/-	4,39	0,40	**10,89**

No match	-/-	4,76	0,45	**10,59**

Stromal cell-derived factor 1 precursor [Source:Uniprot/SWISSPROTAcc:P48061]	-/-	3,94	0,38	**10,36**

ADM precursor [Source:Uniprot/SWISSPROTAcc:P35318]	-/-	3,68	0,36	**10,10**

Beta-2-glycoprotein 1 precursor [Source:Uniprot/SWISSPROTAcc:P02749]	-/-	4,44	0,44	**10,05**

No match	-/-	3,37	0,34	**10,03**

No match	-/-	0,05	5,03	**102,8**

Signal peptide, CUB and EGF-like domain-containing protein 2 precursor [Source:Uniprot/SWISSPROTAcc:Q9NQ36]	-/calcium ion binding	0,09	7,02	**78,01**

No match	-/-	0,18	10,7	**58,98**

Actin, alpha skeletal muscle 1. [Source:Uniprot/SWISSPROTAcc:P68140]	-/structural molecule activity, ATP binding, protein binding	0,44	17,1	**38,39**

No match	-/-	0,05	1,77	**36,79**

No match	-/-	0,09	3,03	**34,37**

No match	-/-	0,45	14,9	**33,47**

Inter-alpha globulIn InhIbItor H3 [Source:IPIAcc:IPI00028413]	hyaluronan metabolic process/serine-type endopeptidase inhibitor activity	0,18	4,41	**24,67**

No match	-/-	0,09	1,92	**21,62**

Hypothetical protein LOC553753 [Source:RefSeq_peptideAcc:NP_001018560]	-/protein binding	0,13	2,84	**21,14**

No match	-/-	0,15	3,18	**20,60**

Tenascin precursor [Source:Uniprot/SWISSPROTAcc:P24821]	signal transduction/receptor binding	0,19	3,87	**20,56**

No match	-/-	0,14	2,77	**20,33**

Prolargin precursor [Source:Uniprot/SWISSPROTAcc:P51888]	-/-	0,16	3,06	**18,89**

Eukaryotic translation initiation factor 4E-1A-binding protein [Source:Uniprot/SWISSPROTAcc:Q98TT6]	negative regulation of translational initiation/eukaryotic initiation factor 4E binding	0,24	2,84	**12,02**

No match	-/-	0,40	4,76	**11,87**

No match	-/-	0,18	2,01	**11,27**

WNT1-inducible-signaling pathway protein 1 precursor [Source:Uniprot/SWISSPROTAcc:O95388]	regulation of cell growth/insulin-like growth factor binding	0,26	2,78	**10,77**

No match	-/	0,29	3,00	**10,34**

Genes were selected according to their patterns of expression (1^st ^group: FC_M _> 1.5 and FC_MV _< 0.67; 2^nd ^group: FC_M _< 0.67 and FC_MV _> 1.5) and highest scores (1^st ^group: FC_M_/FC_MV _> 10; 2^nd ^group: FC_MV_/FC_M _> 10). GO classification was subdivided in biological processes (BP) and molecular function (MF). Two-class SAM test were performed with FDR and FC limits lower than 5% and higher than 1.5.

Finally, proliferative and anti-mineralogenic effects of vanadate suggest that its mechanism of action may be associated with cell differentiation. Therefore, genes that followed i) opposite expression between mineralization and mineralization upon vanadate treatment and ii) concordant expression between vanadate treatments during proliferation and vanadate treatments during mineralization should be of particular interest. A total of 136 genes were differentially expressed when comparing data sets of dividing and mineralizing cell cultures exposed to vanadate. Among those genes, 87 genes were also differentially expressed during *in vitro *mineralization without vanadate (Figure 5B) and genes that fit into the pattern of expression described above were associated to their respective GO categories and listed in Table 3 according to highest FC values. Most genes were associated with metabolism, signaling and cell matrix adhesion. Fewer genes were related to proteolysis, calcium binding, cell cycle and DNA replication.

**Table 3:** Gene description (according to SAPD database [28]), GO classification and FC common genes regulated during mineralization of VSa13 cells (FC_M_) *versus *mineralization + vanadate (FC_MV_) *versus *proliferation + vanadate

Gene description	**GO**_**BP/MF**_	**FC**_**M**_	**FC**_**MV**_	**FC**_**PV**_
Ependymin related protein-1 precursor [Source:IPIAcc:IPI00554718]	cell-matrix adhesion/Ca ion binding	15.21	0.14	0.59

No match	-/-	5.77	0.21	0.28

Xaa-Pro aminopeptidase 2 precursor [Source:Uniprot/SWISSPROTAcc:O43895]	creatine metabolic process, proteolysis/metalloexopeptidase activity, hydrolase activity, creatinase activity	27.2	0.25	0.35

No match	-/-	2.13	0.30	0.24

No match	-/-	3.37	0.34	0.54

Beta-2-glycoprotein 1 precursor [Source:Uniprot/SWISSPROTAcc:P02749]	-/-	4.44	0.44	0.25

Translation initiation factor eIF-2B subunit [Source:Uniprot/SWISSPROTAcc:Q9UI10]	-/-	1.96	0.49	0.58

Rap1b. [Source:Uniprot/SPTREMBLAcc:Q9YH37]	small GTPase mediated signal transduction/GTP binding	2.08	0.51	0.51

Ribonucleoside-diphosphate reductase large subunit [Source:Uniprot/SWISSPROTAcc:P23921]	DNA replication/ribonucleoside-diphosphate reductase activity, protein binding	2.19	0.51	0.66

C9orf119 [Source:Uniprot/SPTREMBLAcc:Q8N2W6]	-/-	2.65	0.53	0.59

No match	-/-	4.00	0.53	0.43

Protein arginine N-methyltransferase 1 [Source:Uniprot/SWISSPROTAcc:Q99873]	-/-	3.90	0.54	0.62

ADP-ribosylation factor 5 [Source:RefSeq_peptideAcc:NP_001653]	small GTPase mediated signal transduction/GTP binding	3.35	0.55	0.61

Argininosuccinate synthase [Source:Uniprot/SWISSPROTAcc:P00966]	arginine biosynthetic process/argininosuccinate synthase activity, ATP binding	3.76	0.55	0.60

Serine/threonine-protein kinase 19. [Source:Uniprot/SWISSPROTAcc:P49842]	-/-	2.25	0.57	0.54

No match	-/-	3.82	0.59	0.53

Phosphate-regulating neutral endopeptidase [Source:Uniprot/SWISSPROTAcc:P78562]	proteolysis/neprilysin activity; metallopeptidase activity, hydrolase activity, Zn ion binding	5.42	0.61	0.39

No match	-/-	2.33	0.61	0.44

Kinetochore-associated protein [Source:Uniprot/SWISSPROTAcc:Q96IY1]	-/	3.20	0.63	0.56

glyoxalase domain containing 5 [Source:RefSeq_peptideAcc:NP_001073958]	-/-	3.81	0.64	0.58

BolA-like protein 2. [Source:Uniprot/SWISSPROTAcc:Q9H3K6]	-/-	1.53	0.66	0.59

Cyclin-dependent kinases regulatory subunit 1 [Source:IPIAcc:IPI00015104]	cell cycle/cyclin-dependent protein kinase regulator activity, kinase activity	3.45	0.66	0.52

Signal peptide, CUB and EGF-like domain-containing protein 2 precursor [Source:Uniprot/SWISSPROTAcc:Q9NQ36]	-/Ca ion binding	0.09	7.02	5.19

No match	-/-	0.05	5.03	2.48

Inter-alpha globulIn InhIbItor H3 [Source:IPIAcc:IPI00028413]	hyaluronan metabolic process/serine-type endopeptidase inhibitor activity	0.18	4.38	2.71

Tenascin precursor [Source:Uniprot/SWISSPROTAcc:P24821]	signal transduction/receptor binding	0.19	3.87	1.55

Prolargin precursor [Source:Uniprot/SWISSPROTAcc:P51888]	-/-	0.16	3.06	2.05

No match	-/-	0.29	3.00	1.82

No match	-/-	0.54	2.76	2.82

No match	-/-	0.29	2.69	2.87

No match	-/-	0.29	2.62	1.75

Thrombospondin 1 precursor [Source:RefSeq_peptideAcc:NP_003237]	cell adhesion/structural molecule activity, Ca ion binding	0.29	2.53	2.28

No match	-/-	0.34	2.14	1.74

No match	-/-	0.51	1.81	2.02

Genes were selected according to their patterns of expression (1^st ^group: FC_M _> 1.5, FC_MV _< 0.67 and FC_PV _< 0.67; 2^nd ^group: FC_M _< 0.67, FC_MV _> 1.5 and FC_PV _> 1.5). GO classification was subdivided in biological processes (BP) and molecular function (MF). Two-class SAM test were performed with FDR and FC limits lower than 5% and higher than 1.5.

### Validation of microarray data by quantitative real-time PCR analysis of gene expression

**Figure 6:**
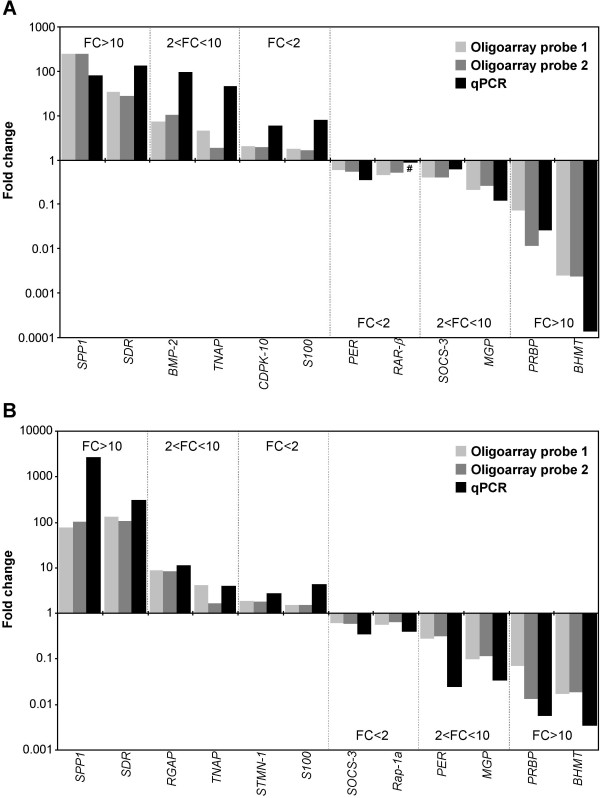
**Real-time PCR analysis of genes detected in VSa13 (A) and VSa16 (B) cells selected for validation**. Genes were selected according to the following criteria: 2 genes with 1.5-2 FC, 2 genes with 2-10 FC, and 2 genes with FC higher than 10. All changes in gene expression evaluated by qPCR were significant according to Student's test (p < 0.05) unless otherwise stated (non-significant - #). Genes selected for analysis were: i) *secreted phosphoprotein 1 (SPP1; osteopontin), photoreceptor outer segment all-trans retinol dehydrogenase (short-chain dehydrogenase reductase; SDR), BMP-2, tissue non-specific alkaline phosphatase (TNAP), cell division protein kinase 10 (CDPK-10), S100 (calcium-binding), periostin precursor (PER), retinoic acid receptor β (RAR-β), suppressor of cytokine signaling 3 (SOCS-3), MGP, plasma retinol-binding protein precursor (PRBP) *and *betaine homocysteine methyltransferase (BHMT) *in VSa13 cells; ii) *SPP1, SDR, Ras GTPase-activating-like protein (RGAP), TNAP, stathmin 1/oncoprotein 18 (STMN-1), S100, SOCS-3, Ras-related protein 1A (Rap-1A), PER, MGP, PRBP *and *BHMT *in VSa16 cells.

Twelve genes differentially expressed during *in vitro *mineralization as for microarray data (6 up- and 6 down-regulated genes) were selected for validation by quantitative real-time PCR (qPCR) according to the following criteria: two genes with FC > 10, two genes with 10 > FC > 2, and two genes with 2 > FC > 1.5 for each cell line. Comparative analysis of microarray and qPCR expression data is presented in Figure 6. Analysis of Pearson correlation revealed coefficients higher than 0.9 for both cell lines (strong correlation) when comparing data from microarray probes 1 and 2, and coefficients between 0.4 and 0.5 for VSa13 cells (moderate correlation) and between 0.5 and 0.8 for VSa16 cells (moderately high correlation) when comparing data from microarray and qPCR. The individual analysis of each gene revealed that FC measured by qPCR were always higher (p < 0.05) than that measured by microarray (with the exception of *RAR-β *in VSa13 cells), suggesting a higher sensitivity of qPCR analysis.

**Figure 7:**
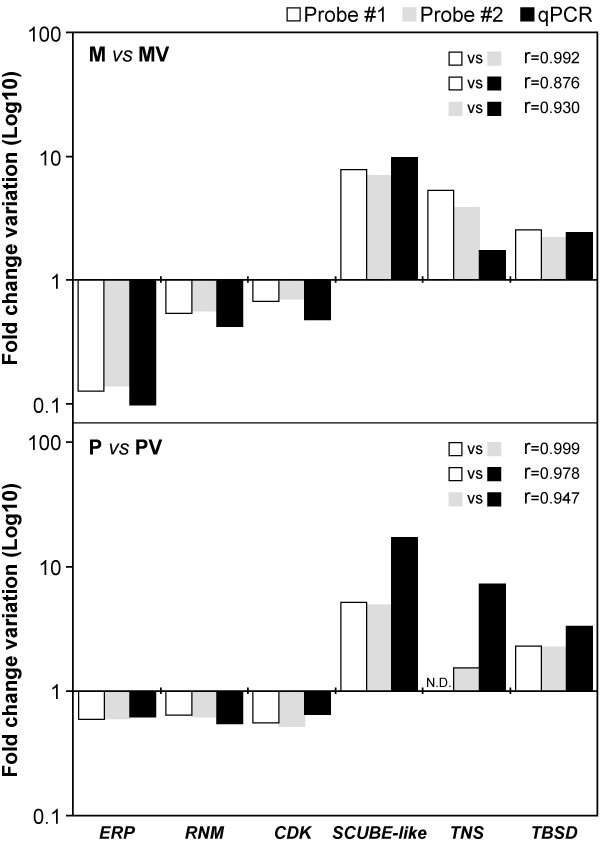
**Real-time PCR analysis of genes detected in vanadate-treated VSa13 cells during mineralization (M *vs *MV) and proliferation (P *vs *PV) selected for validation**. Genes were selected according to the following criteria: 3 up-regulated genes with 1.5-8 FC and 3 down-regulated genes with 1.5-8 FC. All changes in gene expression evaluated by qPCR were significant according to Student's test (p < 0.05). Pearson correlation coefficients, calculated comparing probes 1 and 2 from oligo-array, and comparing probes 1 and 2 from oligo-array with qPCR, are indicated by r. Genes selected for analysis were: *ependymin-related protein (ERP), arginine N-methyltransferase (RNM), cyclin-dependent kinase (CDK), signal peptide CUB and EGF-like protein (SCUBE-like), tenascin (TNS) *and *thrombospondin (TBSD)*.

Similarly, 6 genes differentially expressed in mineralizing cells exposed to vanadate (3 up- and 3 down-regulated genes) were selected for validation of microarray data by qPCR (Figure 7). Analysis of Pearson correlation revealed coefficients higher than 0.99 (strong correlation) when comparing data from microarray probes 1 and 2, and higher than 0.9 (strong correlation) when comparing data from microarray and qPCR. In general, correlation coefficients were shown to be higher among genes analyzed in vanadate-treated VSa13 cells than among those analyzed in mineralizing VSa13 and VSa16 cells. This difference could in part be explained by the lower FC values observed among genes identified in vanadate-treated cells and consequent lower tendency for FC compression of oligo-array data.

## Discussion

### Gilthead seabream oligo-array is a suitable tool to analyze global gene expression of mineralogenic gilthead seabream cell lines

In the present study, a comprehensive set of data has been produced following hybridization of an Agilent 4x44K oligo-array with RNA samples prepared from control, mineralizing and vanadate-treated gilthead seabream vertebra-derived cell samples. A two-class SAM analysis identified mineralogenic genes, some of which had already been previously investigated during *in vitro *mineralization of VSa13 and VSa16 cells through a candidate gene approach, *e.g. tissue non-specific alkaline phosphatase *(*TNAP; *unpublished data), *BMP-2 *[21], *SPP1 *[20], *MGP *and *OC *[19]. At first glance, a good correlation (*i.e. *similar type and extent of regulation) between microarray and pre-existing data was observed. This good correlation was later confirmed through qPCR analysis, although a tendency for FC value compression of oligo-array data was also noted. A similar fold-change compression was observed by Wang et al. while validating two commercial long-oligonucleotide microarray platforms, Applied Biosystems and Agilent, through large scale qPCR analysis of gene expression [29], and more recently by Ferraresso and colleagues while using Agilent gilthead seabream oligo-array [17]. Although our microarray data exhibited a dynamic range inferior to that of qPCR data, a situation which was somehow expected, they were generally accurate; Agilent seabream oligo-array therefore represent a valuable tool for simultaneous analysis of the expression of thousands of genes.

### *In vitro* mineralization recruits similar genes in fish and in mammalian bone-derived systems

Putative mineralogenic genes,* i.e*. those differentially expressed during *in vitro *mineralization of VSa13 and VSa16 cells, presented a similar distribution into GO categories. Approximately half of those genes were shown to be common to VSa13 and VSa16 cells, indicating that both cell lines, although representing different cell types, recruit similar genes and processes during mineralization. Some biological processes were however differentially enriched in both cell lines. In particular, genes related to GO categories *bone mineralization*, *biomineral formation*, *remodeling*, *ossification *and *skeletal development *were enriched in mineralizing VSa16 pre-osteoblast cells, and not in mineralizing VSa13 pre-chondrocyte cells. Another goal of this study was to investigate the conservation of mineralization mechanisms throughout evolution by comparing pattern of gene expression in fish and mammalian bone-derived cell lines. Global analysis of gene expression of ATDC5 cells - mouse pre-chondrocytes similar to VSa13 cells - and MC3T3-E1 cells - mouse pre-osteoblasts similar to VSa16 cells - identified mineralogenic genes associated with catalysis, signal transduction, transport, transcription, structure and motor activity [30] and metabolism, cell cycle, signaling, extracellular matrix, immune response and transcription [31-33], respectively. Similarity in patterns of gene expression of mammalian and fish pre-chondrocyte and pre-osteoblast cell lines, suggested that mechanisms of tissue mineralization might be conserved among vertebrates but also among mineralogenic cell types.

### Anti-mineralogenic activity of vanadate as a way to identify key/novel genes involved in mineralization

Although most of the genes identified in the first step of this analysis certainly play a role during *in vitro *mineralization, some of them must be more important than others. These key genes were identified from the initial bulk of genes by using the anti-mineralogenic activity of vanadate [22,23,34]. Indeed, those genes which expression levels were oppositely regulated during *in vitro *mineralization and upon treatment with vanadate were considered as good candidates. Vanadate stimulates proliferation of VSa13 cells and strongly inhibits its ECM mineralization, and these processes seem to involve MAPK and putative PI-3K\Ras\ERK pathways [22,23]. Genes differentially expressed under these conditions could represent new candidate genes crucial for bone formation. Moreover, vanadium compounds have long been known for their insulin-like properties [35] and role in bone formation [34,36] but to the best of our knowledge, their effects on gene expression have never been investigated, in particular in relation to bone. DAVID functional annotation tool for KEGG pathways identified genes in vanadate-treated VSa13 cells associated with insulin signaling pathway: 3-phosphoinositide-dependent protein kinase-1 in proliferating cells and Ras homolog gene family (member Q) in differentiating cells. The involvement of signaling pathways related to insulin activity is consistent with insulin-mimetic properties of vanadium compounds [35] and recent studies showing that mechanisms of action of vanadate and insulin are similar in fish VSa13 cells and that both molecules exhibit an anti-mineralogenic activity [23]. Among the genes oppositely regulated during *in vitro *mineralization and upon treatment with vanadate (see Table 3), two have been associated to extracellular matrix and previously shown to play an important role in ECM structure: tenascin (normally expressed in mesenchymal stem cells and osteoblasts) and thrombospondin (normally expressed in mesenchymal stem cells and chondrocytes). Both have been associated to fracture healing, spinal curvature and craniofacial defects in knock-out mice [37]. Rap1b, intermediate of MAPK (among other pathways), ADP-ribosylation factor 5, GTP-binding and effector of phospholipase D signaling, and cyclin-dependent kinases regulatory subunit 1, a Ras effector protein, were also among the genes listed in Table 3. Identification of MAPK and Ras intermediate genes further demonstrates the strong involvement of MAPK pathway in the ECM mineralization of bone-derived cells, as recently demonstrated in VSa13 [23] and ATDC5 [38] pre-chondrocyte cells. A *signal peptide CUB and EGF-like protein *(*SCUBE*-like) gene was of particular interest since SCUBE family members have been associated with HH signaling [39], a key pathway in bone formation [1], and were recently shown to modulate/antagonize bone morphogenetic protein activity in transgenic mouse and zebrafish [40,41]. Our data showed an opposite regulation of *SCUBE*-like and *BMP-2 *(associated with bone formation [21,42]) gene expression in mineralizing and vanadate-treated cells, suggesting that SCUBE-like protein may play a key role in anti-mineralogenic activity of vanadate through its action on BMP-2 gene and/or protein. Further studies should however be carried out in order to confirm this hypothesis. Notably, numerous genes detected in this study were classified as unknown. Absence of orthologs in other vertebrate species, high divergence of fish genes and/or low level of annotation in fish sequence databases are likely to contribute to explain this situation. In addition, the fact that numerous genes identified throughout this work have not been previously linked to bone formation, suggests that genetic mechanisms involved in ECM mineralization and bone formation, whether in mammalian or fish species, are still poorly understood.

## Conclusions

Global gene expression has been analyzed during ECM mineralization of gilthead seabream vertebra-derived cell lines using a recently developed oligo-array. A considerably high number of differentially expressed genes was detected, and occurrence of GO categories was found to be similar in both cell lines, with approximately half of the genes common to both cell lines. When comparing occurrence of GO categories in VSa13 and VSa16 with those found in mammalian systems, the similarities found suggested conservation in mineralization-associated processes across vertebrates. Interestingly, enrichment for bone-related genes was observed in VSa16, but not in VSa13, thus reinforcing the previously described association of this cell line to osteoblast lineage. Furthermore, analysis of genes differentially expressed upon exposure to vanadate, a known anti-mineralogenic molecule, has permitted the identification of key/novel mineralogenic genes, which could be classified as: i) annotated genes with known roles in bone formation (e.g. tenascin and thrombospondin), ii) annotated genes with unknown roles in bone formation (e.g. SCUBE-2) and iii) unknown transcripts. Although further analyses are required for genes included in the last two categories, the large number of transcripts detected in this study should bring new insights into the process of mineralization.

## Methods

### Cell culture and ECM mineralization

VSa13 and VSa16 cells were cultured in Dulbecco's modified Eagle medium (DMEM) supplemented with 10% fetal bovine serum, 2 mM L-glutamine, antibiotics and antimycotics (all from Invitrogen), as described previously [19]. ECM mineralization was induced in confluent cultures by supplementing medium with 50 μg/ml of L-ascorbic acid, 10 mM of β-glycerophosphate and 4 mM of calcium chloride (all from Sigma-Aldrich). Culture medium was renewed every 3.5 days. At appropriate times, mineral deposition was evaluated through von Kossa's staining and densitometry analysis [43].

### Preparation of vanadate solution

Vanadate stock solution (5 mM, pH 6.7) was prepared from ammonium metavanadate (Sigma-Aldrich) and stored at 4°C.

### RNA extraction and purification

Total RNA was extracted from cell cultures as described by Chomczynski and Sacchi [44]. RNA samples were purified using QIAGEN RNeasy Mini kit then treated with QIAGEN RNase-free DNase according to manufacturer's instructions. RNA concentration was determined by spectrophotometry (NanoDrop ND-1000, Thermo Scientific) and RNA integrity evaluated by electrophoresis (2100 Bioanalyzer, Agilent Technologies). RNA integrity number (RIN) index was calculated for each sample using Agilent 2100 Expert software. Only RNA samples with a RIN >8 were further processed.

### RNA amplification, labeling and array hybridization

Total RNA (500 ng) was supplemented with a mixture of 10 different viral polyadenylated RNAs (Agilent Spike-In mix) then linearly amplified and labeled with Cy3-dCTP using Agilent One-Color Microarray-Based Gene Expression Analysis protocol. Labeled cRNA was purified using QIAGEN RNeasy Mini kit, and sample concentration and specific activity (pmol Cy3/μg cRNA) were measured by spectrophotometry. Labeled cRNA (1650 ng) was fragmented by adding 11 μl of 10X blocking agent and 2.2 μl of 25X fragmentation buffer, heating at 60°C for 30 min, and finally adding 55 μl of 2X GE hybridization buffer. Hybridization solution (100 μl) was placed in the gasket slide and assembled to the microarray slide (each slide containing four arrays). Slides were incubated for 17 h at 65°C in an Agilent hybridization oven, then dissociated from the hybridization chamber and quickly submerged in GE wash buffer #1 for 1 min. An additional wash was performed in pre-warmed (37°C) GE wash buffer #2 for another 1 min. Slides were scanned using Agilent G2565BA DNA microarray scanner. Scan resolution was set to 5 μm and two different sensitivity levels (XDR Hi 100% and XDR Lo 10%) were used. Both images were analyzed simultaneously using the standard procedures described in Agilent Feature Extraction software 9.5.1.

### Data normalization and statistical analysis

Spots with unsuitable integrity and intensity were filtered out using Feature Extraction Software 9.5.1 flag "glsFound". Flag value is set to 1 if the spot has an intensity value significantly different from the local background, 0 otherwise. Spike-in control intensities (Spike-In Viral RNAs) were used to identify the best normalization procedure for each dataset. After normalization, spike intensities are expected to be uniform across the experiments of a given dataset. Quantile or cyclic Lowess normalization was performed using R statistical software (available at http://www.r-project.org) then SAM statistical test [45] was used to identify differentially expressed genes between groups.

### Analysis of gene expression by quantitative real-time PCR

QPCR was performed using iCycler iQ system (Bio-Rad). Total RNA (1 μg) was treated with RQ1 RNase-free DNase (Promega) then reverse-transcribed at 37°C according to manufacturer's instruction using Moloney murine leukemia virus reverse transcriptase and universal oligo-dT adapter (5'-ACGCGTCGACCTCGAGATCGATG(T)_13_-3'). PCR amplification of cDNA fragments was performed using the iQ SYBR Green I mix, specific primers and 10 ng of reverse-transcribed RNA. The following PCR conditions were used: an initial denaturation step at 95°C for 4 min then 40-50 cycles of amplification (each cycle is 30 s at 95°C, 30 s at 68°C). Fluorescence was measured at the end of each extension cycle in the FAM-490 channel. Levels of gene expression were calculated using the ΔΔCt method and normalized using expression levels of *ribosomal protein L27a *(*RPL27a*) housekeeping gene.

## List of abbreviations

*BMP-2*: *bone morphogenetic protein 2*; DAVID: database for annotation, visualization and integrated discovery; ECM: extracellular matrix; EST: expressed sequence tag; FC: fold change; FDR: false discovery rate; GO: gene ontology; *MGP*: *matrix gla protein*; *OC*: *osteocalcin*; qPCR: quantitative real-time PCR; SAM: significance analysis of microarray; *SCUBE*-like: *signal peptide, CUB and EGF-like protein*; *SPP1*: *secreted phosphoprotein 1; osteopontin*

## Authors' contributions

DMT performed the cell culture experiments, carried out RNA sample preparation and qPCR experiments, participated in oligo-array hybridization, data extraction and data interpretation, and drafted the manuscript. VL participated in the study design and coordination, and helped to draft the manuscript. LB participated in data interpretation and coordination of the study. SF carried out oligo-array hybridization and data extraction, and participated in data interpretation. CR performed the statistical analysis. MLC conceived the study, participated in its design and coordination and contributed to the final draft of the manuscript.

All authors read and approved the final version of the manuscript.

## Supplementary Material

Additional file 1 **Biological processes GO entries occurrence among common differentially expressed genes in control *versus *mineralized VSa13 and VSa16 cells**. Raw data was normalized using quantile method. A two class SAM test was performed; FDR and FC parameters were lower than 5 and higher than 1.5, respectively. Click here for file

Additional file 2 **Molecular function GO entries occurrence among common differentially expressed genes in control *versus *mineralized VSa13 and VSa16 cells**. Raw data was normalized using quantile method. A two class SAM test was performed; FDR and FC parameters were lower than 5 and higher than 1.5, respectively. Click here for file

Additional file 3 **Cellular component GO entries occurrence among common differentially expressed genes in control *versus *mineralized VSa13 and VSa16 cells**. Raw data was normalized using quantile method. A two class SAM test was performed; FDR and FC parameters were lower than 5 and higher than 1.5, respectively. Click here for file

Additional file 4 **Gene description (according to SAPD database **[28]**), GO classification and FC of up-regulated genes in VSa13 cells with FC higher than 10 in control *versus *mineralization**. GO classification was subdivided in biological processes (BP), molecular function (MF) and cellular component (CC). Raw data was normalized using quantile method and then a two class SAM test was performed; FDR was limited to 5%. Click here for file

Additional file 5 **Gene description (according to SAPD database **[28]**), GO classification and FC of up-regulated genes in VSa16 cells with FC higher than 10 in control *versus *mineralization**. GO classification was subdivided in biological processes (BP), molecular function (MF) and cellular component (CC). Raw data was normalized using quantile method and then a two class SAM test was performed; FDR was limited to 5%. Click here for file

Additional file 6 **Gene description (according to SAPD database **[28]**), GO classification and FC of down-regulated genes in VSa13 cells with FC higher than 10 in control *versus *mineralization**. GO classification was subdivided in biological processes (BP), molecular function (MF) and cellular component (CC). Raw data was normalized using quantile method and then a two class SAM test was performed; FDR was limited to 5%. Click here for file

Additional file 7 **Gene description (according to SAPD database **[28]**), GO classification and FC of down-regulated genes in VSa16 cells with FC higher than 10 in control *versus *mineralization**. GO classification was subdivided in biological processes (BP), molecular function (MF) and cellular component (CC). Raw data was normalized using quantile method and then a two class SAM test was performed; FDR was limited to 5%. Click here for file
