# Supplementary material for: Global analysis of gene expression in mineralizing fish vertebra-derived cell lines: new insights into anti-mineralogenic effect of vanadate
Source: BMC Genomics. 2011 Jun 13;12:310. doi: 10.1186/1471-2164-12-310 (PMC3141667; doi:10.1186/1471-2164-12-310)
Supplement: Additional file 1 — Biological processes GO entries occurrence among common differentially expressed genes in control versus mineralized VSa13 and VSa16 cells. Raw data was normalized using quantile method. A two class SAM test was performed; FDR and FC parameters were lower than 5 and higher than 1.5, respectively. [file 1471-2164-12-310-S1.DOC]

**Additional file 1 – Additional table S1 ‑ Biological processes GO entries occurrence among common differentially expressed genes in control *versus* mineralized VSa13 and VSa16 cells.** Raw data was normalized using quantile method. A two class SAM test was performed; FDR and FC parameters were lower than 5 and higher than 1.5, respectively.

| **Biological process description** | **Occurrence (%)** |
| --- | --- |
| Metabolic process | 54.4 |
| Cellular metabolic process (MAPK activation, glycogen synthesis, glycolysis, CAT cycle; nucleobase, nucleoside, nucleotide and nucleic acid metabolism; monosaccharide, phosphate, amino acid and derivative, sulfur, one-carbon compound, cofactor, superoxide, vitamin and carboxylic acid metabolism) | 19.0 |
| Macromolecule metabolic process (ubiquitin-dependent catabolism; RNA and DNA metabolism: modification, replication, recombination, translation and processing; protein metabolism: folding, modification, cross-linking and proteolysis) | 15.6 |
| Primary metabolic process (carbohydrate and lipid) | 5.4 |
| Biosynthetic process (gluconeogenesis, carbohydrate, Fe-S cluster, GSH, isoprenoid...) | 0.8 |
| General metabolic process and others | 8.0 |
| Establishment of localization | 18.8 |
| Transport (ion, lipid, proton) | 14.9 |
| Establishment of localization in cell (protein: targeting, import into nucleus and intracellular transport) | 3.9 |
| Cellular process | 13.2 |
| Cell communication (signalling) | 6.8 |
| Cellular component organization and biogenesis (chromatin, cytoskeleton, ribosome) | 2.0 |
| Others (redox homeostasis, cell cycle, apoptosis, ATP synthesis, transposition; nucleosome assembly) | 4.4 |
| Biological regulation | 7.1 |
| Regulation of biological process (cell cycle, growth, cell death, biosynthesis, signal transduction; gene expression: transcription, translation and DNA methylation) | 7.1 |
| Biological adhesion | 2.2 |
| Cell adhesion (cell-cell, cell-matrix…) | 2.2 |
| Response to stimulus | 1.7 |
| Stress and chemical (DNA repair, oxidative stress…) | 1.7 |
| Others | 2.7 |
| Immune system, developmental process, macromolecular complex subunit organization, multicellular organismal process | 2.7 |
