# Supplementary material for: Global analysis of gene expression in mineralizing fish vertebra-derived cell lines: new insights into anti-mineralogenic effect of vanadate
Source: BMC Genomics. 2011 Jun 13;12:310. doi: 10.1186/1471-2164-12-310 (PMC3141667; doi:10.1186/1471-2164-12-310)
Supplement: Additional file 2 — Molecular function GO entries occurrence among common differentially expressed genes in control versus mineralized VSa13 and VSa16 cells. Raw data was normalized using quantile method. A two class SAM test was performed; FDR and FC parameters were lower than 5 and higher than 1.5, respectively. [file 1471-2164-12-310-S2.DOC]

**Additional file 2 – Additional table S2 –** **Molecular function GO entries occurrence among common differentially expressed genes in control *versus* mineralized VSa13 and VSa16 cells.** Raw data was normalized using quantile method. A two class SAM test was performed; FDR and FC parameters were lower than 5 and higher than 1.5, respectively.

| **Molecular function description** | **Occurrence (%)** |
| --- | --- |
| Binding | 43.6 |
| Nucleotide (ATP, GTP…) | 16.0 |
| Ion (metal: Fe, Zn, Cu, Ca, Mg) | 8.2 |
| Protein (receptor, IGF, calmodulin, TIF, cytoskeletal, transmembrane transporter…) | 8.0 |
| Nucleic acid (RNA, DNA) | 7.8 |
| General protein binding | 3.6 |
| Catalytic activity | 41.0 |
| Transferase (kinase, phospho, gamma-glutamyl, acetylglucosaminyl, N-acetyl, ribosyl, aminomethyl, S-methyl, O-methyl, glutathione…) | 10.4 |
| Hydrolase (phosphatase: AP, ALP, PTP, PIP; peptidase, GTPase, arginase, nuclease) | 8.8 |
| Oxidoreductase (oxidase: e.g. cyt-c; dehydrogenase: e.g. G6PDH, NADH; reductase: e.g. GSSG, cyt-c; oxygenase; desaturase; SOD) | 7.6 |
| General catalytic activity (isomerase, ligase, lyase, cyclase and deaminase) | 14.2 |
| Transporter activity | 4.6 |
| Transmembrane transporter (voltage-gated and passive channel, Na+/K+ ATPase, proton ATP synthase | 1.6 |
| General transporter activity | 3.0 |
| Enzyme regulator activity | 3.6 |
| Enzyme inhibitor (protease inhibitor…) | 1.2 |
| GTPase regulator (ARF and Ras regulator...) | 1.2 |
| Enzyme activator (GTPase activator…) | 0.8 |
| Proteasome activator | 0.4 |
| Molecular transducer activity | 2.4 |
| Signal transducer (transmembrane receptor) | 2.4 |
| Others | 4.8 |
| Structural molecule, motor, antioxidant, electron carrier, transcription and translation regulator | 4.8 |
