# Supplementary material for: Global analysis of gene expression in mineralizing fish vertebra-derived cell lines: new insights into anti-mineralogenic effect of vanadate
Source: BMC Genomics. 2011 Jun 13;12:310. doi: 10.1186/1471-2164-12-310 (PMC3141667; doi:10.1186/1471-2164-12-310)
Supplement: Additional file 3 — Cellular component GO entries occurrence among common differentially expressed genes in control versus mineralized VSa13 and VSa16 cells. Raw data was normalized using quantile method. A two class SAM test was performed; FDR and FC parameters were lower than 5 and higher than 1.5, respectively. [file 1471-2164-12-310-S3.DOC]

**Additional file 3 – Additional table S3 – Cellular component GO entries occurrence among common differentially expressed genes in control *versus* mineralized VSa13 and VSa16 cells.** Raw data was normalized using quantile method. A two class SAM test was performed; FDR and FC parameters were lower than 5 and higher than 1.5, respectively.

| **Cellular component description** | **Occurrence (%)** |
| --- | --- |
| Cell part | 59.0 |
| Intracellular or intracellular part (cytosol, mitochondrion) | 32.7 |
| Membrane or membrane part (plasma membrane, ER) | 24.3 |
| Cell fraction (soluble, insoluble vesicular) | 1.2 |
| Periplasmic space | 0.8 |
| Organelle | 13.9 |
| Intracellular organelle (nucleus, ER, Golgi) | 13.5 |
| Non-membrane-bounded organelle (cytoskeleton) | 0.4 |
| Macromolecular complex | 10.0 |
| Protein complex (myosin, proteasome, microtubule associated…) | 7.2 |
| Protein-DNA complex (nucleosome) | 1.6 |
| Ribonucleoprotein complex | 0.8 |
| Protein-lipid complex (plasma lipoprotein particle) | 0.4 |
| Extracellular | 8.4 |
| Extracellular region | 7.6 |
| Extracellular matrix (proteinaceous) | 0.8 |
| Organelle part | 7.2 |
| Intracellular organelle part (chromosomal, cytoskeletal, Golgi vesicle) | 5.2 |
| Organelle membrane (Golgi, inner and outer mitochondrial) | 2.0 |
| Envelope | 1.6 |
| Organelle envelope (mitochondrial) | 1.6 |
