# Supplementary material for: Global analysis of gene expression in mineralizing fish vertebra-derived cell lines: new insights into anti-mineralogenic effect of vanadate
Source: BMC Genomics. 2011 Jun 13;12:310. doi: 10.1186/1471-2164-12-310 (PMC3141667; doi:10.1186/1471-2164-12-310)
Supplement: Additional file 4 — Gene description (according to SAPD database [28]), GO classification and FC of up-regulated genes in VSa13 cells with FC higher than 10 in control versus mineralization. GO classification was subdivided in biological processes (BP), molecular function (MF) and cellular component (CC). Raw data was normalized using quantile method and then a two class SAM test was performed; FDR was limited to 5%. [file 1471-2164-12-310-S4.DOC]

**Additional file 4 – Additional table S4 – Gene description (according to SAPD database [28]), GO classification and FC of up‑regulated genes in VSa13 cells with FC higher than 10 in control *versus* mineralization.** GO classification was subdivided in biological processes (BP), molecular function (MF) and cellular component (CC). Raw data was normalized using quantile method and then a two class SAM test was performed; FDR was limited to 5%.

| **Gene description** | **GO (BP/ MF/ CC)** | **FC** |
| --- | --- | --- |
| SPP1 (osteopontin) [NP001002308] | - /- / plasma membrane part | 247.9 |
| Hypothetical FGF binding protein [NP_001082995] | - / - / - | 116.8 |
| No match | - / - / - | 71.0 |
| AMBP protein precursor [P02760] | - / serine-type endopeptidase inhibitor activity / - | 47.4 |
| Na/K ATPase 1 [P05023] | - / ATPase activity coupled to transmembrane movement of cations / integral to membrane | 40.2 |
| No match | - / - / - | 35.3 |
| Photoreceptor outer segment all-trans retinol dehydrogenase (SDR) [IPI00024598] | metabolic process / oxidoreductase activity / - | 34.8 |
| Hypothetical actinoporin-like protein [Danio rerio] | cation transport / channel activity / pore complex | 30.0 |
| No match | - / - / - | 28.2 |
| Xaa-Pro aminopeptidase 2 precursor [O43895] | creatine metabolic process, proteolysis / metalloexopeptidase activity, creatinase activity / - | 27.2 |
| No match | - / - / - | 25.5 |
| Hypothetical vitellogenin 2 isoform 1 [NP_001038378] | lipid transport / lipid transporter activity / - | 24.1 |
| No match | - / - / - | 22.2 |
| No match | - / - / - | 20.2 |
| Multidrug resistance-associated protein 1 [P33527] | transport / ATPase transporter activity / integral to membrane | 19.1 |
| Hypothetical protein [Danio rerio] | cation transport / channel activity / pore complex | 18.3 |
| No match | - / - / - | 17.5 |
| No match | - / - / - | 17.0 |
| No match | - / - / - | 17.0 |
| No match | - / - / - | 16.6 |
| Homolog of Homo sapiens ependymin related protein-1 [IPI00554718] | cell-matrix adhesion / Ca ion binding / extracellular region | 15.2 |
| Matrix metalloproteinase-9 precursor [P14780] | metabolic process, proteolysis / metalloendopeptidase activity, Ca and Zn ion binding / proteinaceous extracellular matrix | 15.0 |
| No match | - / - / - | 14.0 |
| Prostaglandin D synthase [Q8QGV4] | lipid metabolic process, transport / transporter activity / - | 13.9 |
| No match | - / - / - | 13.8 |
| No match | - / - / - | 13.8 |
| No match | - / - / - | 13.1 |
| No match | - / - / - | 13.1 |
| Guanine nucleotide-binding protein [P59768] | G-protein coupled receptor protein / signal transducer activity / heterotrimeric G-protein complex | 12.8 |
| No match | - / - / - | 12.7 |
| No match | - / - / - | 12.6 |
| Microtubule-associated proteins [Q9BXW4] | - / - / - | 12.6 |
| No match | - / - / - | 12.3 |
| No match | - / - / - | 12.0 |
| No match | - / - / - | 11.8 |
| KIAA1199 [NP_061159] | - / sugar binding / - | 11.7 |
| TNF superfamily member 14 [O43557] | immune response / TNF receptor binding / membrane | 11.5 |
| No match | - / - / - | 11.2 |
| No match | - / - / - | 11.2 |
| No match | - / - / - | 11.1 |
| No match | - / - / - | 11.1 |
| Fatty acid desaturase 2 [NP_004256] | - / oxidoreductase activity / - | 10.8 |
| No match | - / - / - | 10.7 |
| No match | - / - / - | 10.6 |
| No match | - / - / - | 10.6 |
| Bone morphogenetic protein 2 (BMP-2) [P12643] | - / growth factor activity / extracellular region | 10.5 |
