# Supplementary material for: Global analysis of gene expression in mineralizing fish vertebra-derived cell lines: new insights into anti-mineralogenic effect of vanadate
Source: BMC Genomics. 2011 Jun 13;12:310. doi: 10.1186/1471-2164-12-310 (PMC3141667; doi:10.1186/1471-2164-12-310)
Supplement: Additional file 5 — Gene description (according to SAPD database [28]), GO classification and FC of up-regulated genes in VSa16 cells with FC higher than 10 in control versus mineralization. GO classification was subdivided in biological processes (BP), molecular function (MF) and cellular component (CC). Raw data was normalized using quantile method and then a two class SAM test was performed; FDR was limited to 5%. [file 1471-2164-12-310-S5.DOC]

**Additional file 5 – Additional table S5 – Gene description (according to SAPD database [28]), GO classification and FC of up‑regulated genes in VSa16 cells with FC higher than 10 in control *versus* mineralization.** GO classification was subdivided in biological processes (BP), molecular function (MF) and cellular component (CC). Raw data was normalized using quantile method and then a two class SAM test was performed; FDR was limited to 5%.

| **Gene description** | **GO (BP/ MF/ CC)** | **FC** |
| --- | --- | --- |
| No match | - / - / - | 1653.9 |
| Na/K ATPase 1 [P05023] | - / ATPase activity, transmembrane movement of Ca ions / integral to membrane | 643.3 |
| Hypothetical protein similar to apolipoprotein C1 | - / - / - | 195.8 |
| No match | - / - / - | 182.6 |
| AMBP protein precursor [P02760] | - / serine-type endopeptidase inhibitor activity / - | 150.0 |
| No match |  | 117.9 |
| Photoreceptor outer segment all-trans retinol dehydrogenase (SDR) [IPI00024598] | metabolic process / oxidoreductase activity / - | 108.0 |
| SPP1 (osteopontin) [NP_001002308] | - / - / plasma membrane part | 101.1 |
| No match | - / - / - | 53.8 |
| No match | - / - / - | 49.4 |
| No match | - / - / - | 47.6 |
| No match | - / - / - | 39.3 |
| Hypothetical actinoporin-like protein [Danio rerio] | - / - / - | 32.3 |
| No match | - / - / - | 27.6 |
| Na:K-transporting ATPase subunit ß-1 [P05026] | - / Na:K-exchanging ATPase activity / integral to membrane | 25.6 |
| No match | - / - / - | 25.5 |
| Leukocyte surface antigen CD53 [P19397] | - / - / integral to membrane | 25.2 |
| No match | - / - / - | 21.7 |
| Putative protein serine/threonine kinase | - / - / - | 21.2 |
| No match | - / - / - | 21.1 |
| Prostaglandin D synthase [Q8QGV4] | lipid metabolic process/ transporter activity / - | 20.9 |
| No match | - / - / - | 20.2 |
| Similar to ribosomal protein L7 [NP000962.2] | - / - / - | 19.4 |
| No match | - / - / - | 19.0 |
| Similar to unnamed protein | - / - / - | 18.4 |
| No match | - / - / - | 18.3 |
| Connector enhancer of kinase suppressor of ras 1 [Q969H4] | - / - / - | 17.4 |
| No match | - / - / - | 15.0 |
| No match | - / - / - | 14.7 |
| Fasciculation / elongation protein Z1 [Q99689] | - / - / - | 14.7 |
| No match | - / - / - | 13.4 |
| S100-A2 protein [P29034] | - / Ca ion binding / - | 12.9 |
| No match | - / - / - | 12.7 |
| No match | - / - / - | 12.7 |
| No match | - / - / - | 11.8 |
| No match | - / - / - | 11.6 |
| C-X-C chemokine receptor type 7 [P25106] | - / G-protein coupled receptor protein signalling pathway / rhodopsin-like receptor activity / integral to membrane | 11.5 |
| Radical S-adenosyl methionine domain containing 2 [NP_542388] | - / catalytic activity, Fe-S cluster binding / - | 11.4 |
| No match | - / - / - | 11.3 |
| No match | - / - / - | 11.1 |
| No match | - / - / - | 11.1 |
| No match | - / - / - | 10.8 |
| No match | - / - / - | 10.8 |
| No match | - / - / - | 10.7 |
| No match | - / - / - | 10.7 |
| No match | - / - / - | 10.3 |
| No match | - / - / - | 10.2 |
| No match | - / - / - | 10.0 |
