# Supplementary material for: Global analysis of gene expression in mineralizing fish vertebra-derived cell lines: new insights into anti-mineralogenic effect of vanadate
Source: BMC Genomics. 2011 Jun 13;12:310. doi: 10.1186/1471-2164-12-310 (PMC3141667; doi:10.1186/1471-2164-12-310)
Supplement: Additional file 6 — Gene description (according to SAPD database [28]), GO classification and FC of down-regulated genes in VSa13 cells with FC higher than 10 in control versus mineralization. GO classification was subdivided in biological processes (BP), molecular function (MF) and cellular component (CC). Raw data was normalized using quantile method and then a two class SAM test was performed; FDR was limited to 5%. [file 1471-2164-12-310-S6.DOC]

**Additional file 6 – Additional table S6 – Gene description (according to SAPD database [28]), GO classification and FC of down‑regulated genes in VSa13 cells with FC higher than 10 in control *versus* mineralization.** GO classification was subdivided in biological processes (BP), molecular function (MF) and cellular component (CC). Raw data was normalized using quantile method and then a two class SAM test was performed; FDR was limited to 5%.

| **Gene description** | **GO (BP/ MF/ CC)** | **FC** |
| --- | --- | --- |
| Betaine-homocysteine methyltransferase (BHMT) [Q5PSM1] | - / betaine-homocysteine S-methyltransferase activity / - | 417.0 |
| No match | - / - / - | 132.3 |
| Angiopoietin-related protein 4 [Q9BY76] | - / - / - | 101.9 |
| Plasma retinol-binding protein (PRBP) [P02753] | transport / transporter activity, retinoid binding / - | 85.1 |
| Dimethylglycine dehydrogenase [Q9UI17] | glycine catabolic process, choline metabolic process / aminomethyltransferase activity, folic acid binding, oxidoreductase activity, dimethylglycine dehydrogenase activity, FAD binding / cytoplasm, mitochondrion | 76.3 |
| Homolog of Oryzias latipes Alcohol dehydrogenase Class VI. [Q6B4J3] | - / Zn ion binding, oxidoreductase activity / - | 46.5 |
| Angiopoietin-related protein 7 [O43827] | - / signal transduction, receptor binding / - | 39.5 |
| Fructose-bisphosphate aldolase P05062] | glycolysis / catalytic activity, lyase activity / - | 34.7 |
| No match | - / - / - | 31.1 |
| Similar to Homo sapiens tetratricopeptide repeat domain 36 [NM_001080441] | - / binding / - | 30.6 |
| No match | - / - / - | 30.5 |
| No match | - / - / - | 26.9 |
| No match | - / - / - | 24.1 |
| No match | - / - / - | 21.8 |
| No match | - / - / - | 21.6 |
| Glucose-6-phosphate isomerase [P06744] | gluconeogenesis, glycolysis / glucose-6-phosphate isomerase activity / - | 21.3 |
| No match | - / - / - | 20.8 |
| No match | - / - / - | 20.4 |
| No match | - / - / - | 19.9 |
| No match | - / - / - | 17.2 |
| Sulfotransferase family 1 [Q6DHG7] | - / sulfotransferase activity / - | 16.9 |
| No match | - / - / - | 16.4 |
| Myelin basic protein [P02686] | - / - / - | 16.4 |
| No match | - / - / - | 16.3 |
| Phosphomannomutase 1 [Q92871] | - / - / - | 16.2 |
| No match | - / - / - | 15.9 |
| No match | - / - / - | 15.0 |
| No match | - / - / - | 14.9 |
| No match | - / - / - | 14.8 |
| Hypothetical S100v2 [NP_001073630] | - / Ca ion binding / - | 14.4 |
| No match | - / - / - | 14.2 |
| No match | - / - / - | 13.9 |
| No match | - / - / - | 13.1 |
| No match | - / - / - | 13.0 |
| No match | - / - / - | 12.3 |
| No match | - / - / - | 12.2 |
| No match | - / - / - | 12.2 |
| No match | - / - / - | 11.6 |
| No match | - / - / - | 11.4 |
| No match | - / - / - | 11.2 |
| Similar to uncharacterized protein | - / - / - | 11.2 |
| Signal peptide, CEGP1. [Q9NQ36] | - / Ca ion binding / - | 11.1 |
| No match | - / - / - | 10.9 |
| No match | - / - / - | 10.7 |
| Similar to unnamed protein | - / - / - | 10.6 |
| No match | - / - / - | 10.6 |
| No match | - / - / - | 10.4 |
| Kainate receptor beta subunit. [Q90278] | ion transport / extracellular-glutamate-gated ion channel activity / integral to membrane, cell junction, postsynaptic membrane | 10.2 |
| ERO1-like [NP_956644] | protein folding / protein thiol-disulfide exchange, unfolded protein response, protein binding, oxidoreductase activity, FAD binding / endoplasmic reticulum membrane | 10.1 |
