# Supplementary material for: Global analysis of gene expression in mineralizing fish vertebra-derived cell lines: new insights into anti-mineralogenic effect of vanadate
Source: BMC Genomics. 2011 Jun 13;12:310. doi: 10.1186/1471-2164-12-310 (PMC3141667; doi:10.1186/1471-2164-12-310)
Supplement: Additional file 7 — Gene description (according to SAPD database [28]), GO classification and FC of down-regulated genes in VSa16 cells with FC higher than 10 in control versus mineralization. GO classification was subdivided in biological processes (BP), molecular function (MF) and cellular component (CC). Raw data was normalized using quantile method and then a two class SAM test was performed; FDR was limited to 5%. [file 1471-2164-12-310-S7.DOC]

**Additional file 7 – Additional table S7 – Gene description (according to SAPD database [28]), GO classification and FC of down‑regulated genes in VSa16 cells with FC higher than 10 in control *versus* mineralization.** GO classification was subdivided in biological processes (BP), molecular function (MF) and cellular component (CC). Raw data was normalized using quantile method and then a two class SAM test was performed; FDR was limited to 5%.

| **Gene description** | **GO (BP/ MF/ CC)** | **FC** |
| --- | --- | --- |
| Alcohol dehydrogenase Class VI [Q6B4J3] | - / Zn ion binding, oxidoreductase activity / - | 221.3 |
| No match | - / - / - | 121.5 |
| Angiopoietin-related protein 7 [O43827] | signal transduction / receptor binding / - | 79.1 |
| Plasma retinol-binding protein (PRBP) [P02753] | transport / transporter activity, retinoid binding / - | 76.4 |
| Similar to normal mucosa of esophagus‑specific gene 1 | - / - / - | 73.4 |
| No match | - / - / - | 67.7 |
| Fibulin 5 | - / Ca ion binding / - | 66.4 |
| Kainate receptor beta subunit. [Q90278] | ion transport /extracellular-glutamate-gated ion channel / integral to membrane, postsynaptic membrane, cell junction | 66.1 |
| Betaine-homocysteine methyltransferase (BHMT) [Q5PSM1] | betaine-homocysteine S-methyltransferase activity | 58.2 |
| No match | - / - / - | 53.8 |
| Similar to unnamed protein | - / - / - | 34.5 |
| No match | - / - / - | 32.0 |
| No match | - / - / - | 29.4 |
| No match | - / - / - | 29.2 |
| Actin, alpha skeletal muscle 1 [P68140] | - / structural molecule activity, ATP binding, protein binding / cytoplasm, cytoskeleton | 28.1 |
| Similar to unnamed protein | - / - / - | 23.6 |
| No match | - / - / - | 22.9 |
| No match | - / - / - | 21.7 |
| No match | - / - / - | 21.7 |
| No match | - / - / - | 21.2 |
| No match | - / - / - | 20.4 |
| Glucose-6-phosphate isomerase [P06744] | gluconeogenesis, glycolysis / glucose-6-phosphate isomerase activity / - | 20.4 |
| No match | - / - / - | 20.2 |
| Similar to male-specific protein | - / - / - | 17.2 |
| Interferon induced protein 2 [Q8JH61] | response to biotic stimulus / - / integral to membrane | 16.0 |
| No match | - / - / - | 15.9 |
| Troponin T [P13805] | - / - / - | 15.0 |
| Cytosolic sulfotransferase 3 [Q6DHG7] | - / sulfotransferase activity / - | 15.0 |
| Angiopoietin-related protein 4 [Q9BY76] | signal transduction / receptor binding / extracellular | 14.4 |
| No match | - / - / - | 14.0 |
| Heat-shock protein ß-7 [Q9UBY9] | - / - / - | 13.9 |
| No match | - / - / - | 13.7 |
| EGF-like domain-containing protein 7 [Q9UHF1] | - / - / - | 13.6 |
| No match | - / - / - | 13.5 |
| No match | - / - / - | 13.5 |
| No match | - / - / - | 13.1 |
| No match | - / - / - | 13.0 |
| No match | - / - / - | 12.2 |
| No match | - / - / - | 12.2 |
| ERO1-like [NP_956644] | protein folding / protein thiol-disulfide exchange, unfolded protein response, protein binding, oxidoreductase activity, FAD binding / endoplasmic reticulum membrane | 11.9 |
| No match | - / - / - | 11.9 |
| No match | - / - / - | 11.4 |
| Similar to tetratricopeptide repeat 36 | - / binding / - | 11.3 |
| Hepcidin-like. [Q6Q5X4] | - / - / - | 10.9 |
| No match | - / - / - | 10.8 |
| No match | - / - / - | 10.5 |
| SDR12 isoform 2 [NP_078981] | metabolic process / oxidoreductase activity / - | 10.3 |
| Matrix gla protein (MGP) [P08493] | - / Ca ion binding / extracellular region | 10.3 |
| No match | - / - / - | 10.2 |
